# Predictors and benefits of lipid-lowering therapy initiation after an atherosclerotic cardiovascular event: a retrospective cohort study

**DOI:** 10.3389/fphar.2025.1588376

**Published:** 2025-09-25

**Authors:** Manuela Casula, Stefano Scotti, Federica Galimberti, Giacomo Matteo Bruno, Giorgio Lorenzo Colombo, Asiiat Alieva, Sining Xie, Alberico Luigi Catapano, Elena Olmastroni

**Affiliations:** ^1^ Epidemiology and Preventive Pharmacology Service (SEFAP), Department of Pharmacological and Biomolecular Sciences, University of Milan, Milan, Italy; ^2^ IRCCS MultiMedica, Sesto S. Giovanni, Milan, Italy; ^3^ Center of Research, S.A.V.E. Studi-Health Economics and Outcomes Research, Milan, Italy; ^4^ Department of Drug Sciences, University of Pavia, Pavia, Italy; ^5^ Research laboratory of lipid disorders and atherosclerosis, Almazov National Medical Research Centre, Saint Petersburg, Russia

**Keywords:** lipid-lowering therapies, atherosclerotic cardiovascular event, secondary prevention, guidelines compliance, treatment outcomes

## Abstract

**Background:**

Guidelines recommend lipid-lowering therapy (LLT) after an atherosclerotic cardiovascular disease (ASCVD) event. This study investigated real-world LLT initiation rate and its effect on total mortality in the Lombardy region.

**Methods:**

Individuals aged ≥40 with an ASCVD event between January and September 2022 were identified from Lombardy’s administrative data. The prevalence of LLT initiation within 3 months was estimated, and factors influencing treatment initiation were evaluated using multivariate logistic regression (odds ratios [OR] and 95% confidence intervals [95% CI]). One-year post-event mortality was analyzed.

**Results:**

Among 16,025 patients 41.14% did not receive a LLT after an ASCVD event. Treatment initiation was more likely in subjects hospitalized for a cardiovascular event (OR 2.22, 95%CI 2.07–2.38, vs. cerebrovascular event), in patients aged 51–60 years (OR 1.30, 95%CI 1.16–1.46), and in patients previously treated with antidiabetic (OR 1.42, 95%CI 1.25–1.62), antihypertensive (OR 1.96, 95%CI 1.80–2.13), and thyroid hormone replacement medications (OR 1.34, 95%CI 1.10–1.63). Conversely, older age (71–80 years: OR 0.79, 95%CI 0.71–0.87; >80 years: OR 0.47, 95%CI 0.42–0.52), female sex (OR 0.73, 95%CI 0.68–0.79), previous exposure to antithrombotic medications (OR 0.65, 95%CI 0.59–0.72), and polypharmacy (OR 0.90, 95%CI 0.81–0.99 for 5-9 medications, OR 0.61, 95%CI 0.52–0.72 for ≥10 medications) reduced the likelihood of treatment. Mortality at 1 year was 3.07% in treated versus 11.66% in untreated patients (p-value <0.001).

**Conclusion:**

This study underscores a suboptimal LLT initiation rate in post-ASCVD patients. Initiating LLT is associated with significantly reduced 1-year total mortality, highlighting the need to optimize secondary prevention strategies.

## 1 Introduction

Global death counts due to cardiovascular disease (CVD) increased from 12.4 million in 1990 to 19.8 million in 2022, with ischemic heart disease representing the leading cause of cardiovascular mortality worldwide (Mensah et al., 2023). In Italy (Saglietto et al., 2021), the crude incidence of CVD is nearly twice as high as the global prevalence (12.9% vs. 6.6%). The incidence rates are even higher in individuals who have already developed an atherosclerotic cardiovascular disease (ASCVD) event, particularly during the first 6 months after the index event (Jernberg et al., 2015; Galasso et al., 2021). For these individuals, lipid management should be integrated into a global risk management approach (Mach et al., 2020). Statins are the cornerstone of drug therapy for low-density lipoprotein cholesterol (LDL-C) management (Baigent et al., 2005). The use of statins after an ASCVD event reduces the 1-year risk of a new event by approximately 22%, with the effect being even more pronounced the earlier the therapy is started (Tecson et al., 2022). In subjects who require more substantial reductions in LDL-C to achieve treatment goals, guidelines recommend combination therapies (Masana et al., 2023) with ezetimibe (Olmastroni et al., 2024) and, more recently, the use of additional strategies such as proprotein convertase subtilisin/kexin type 9 (PCSK9) inhibition (Board et al., 2020). Beyond therapeutic strategies, the European Society of Cardiology guidelines (Byrne et al., 2023) strongly advocate for the early initiation of lipid-lowering therapy (LLT) to achieve maximum prognostic benefit. Even though the importance of starting treatment immediately and maintaining adherence to it is well established, observational studies have revealed that the LLT use in the secondary prevention of ASCVD events remains suboptimal (Banach and Penson, 2020; Tecson et al., 2022).

Our aim was to provide updated data on the LLT prescription in a large Italian cohort of patients discharged from hospital after an ASCVD event, assessing demographic and clinical factors associated with higher likelihood of receiving LLT, as well as the benefit, in terms of all-cause mortality, of initiating any LLT within 1 year following the first hospitalization.

## 2 Methods

### 2.1 Data source

Data were retrieved from the healthcare utilization databases of Lombardy Region (data availability 2017–2023), in detail: 1) the archive of Lombardy’s residents with a coverage from the Italian National Health Service (NHS), containing demographic variables (sex, date of birth, date of death); 2) the drugs’ prescription archive, including information on the drugs reimbursed by NHS delivered from any pharmacy in the Region, as the corresponding Anatomical Therapeutic Chemical (ATC) code and the prescription date, and 3) the hospital discharge archive recording, among others, the admission and discharge dates and primary and secondary diagnoses of all hospitalizations at public or private hospitals of the Region.

The data belonging to each subject listed in different archives are linkable using a subject’s unique encrypted identification key, allowing the full medical history of each individual belonging to the target population to be retrieved. The identification key was appropriately encrypted to prevent the identification of the subjects, as for the European Regulation No. 2016/679 and the national Legislative Decree 101/2018.

### 2.2 Study population

**FIGURE 1:**
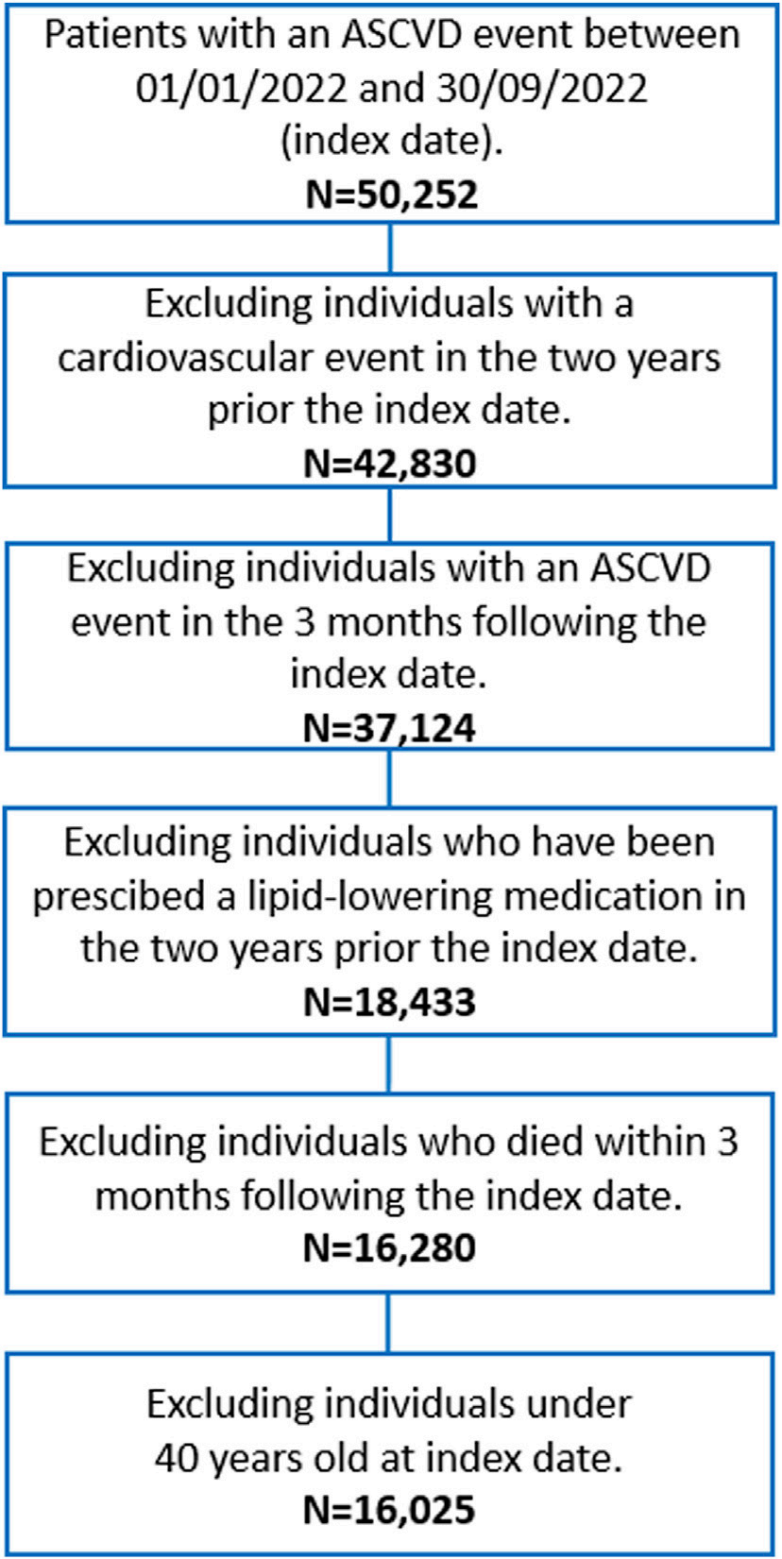
Flow chart of patient selection.

The flow chart of patient selection is depicted in Figure 1. All beneficiaries of the NHS, resident in Lombardy, of both sexes, with age ≥40 years, who were hospitalised for an ASCVD event between January 1, 2022, and September 30, 2022 were included in the cohort. We searched for International Classification of Diseases, Ninth Revision, diagnosis code “410, 411, 413, 414” for coronary disease and “433, 434, 435” for ischemic cerebrovascular disease (haemorrhagic events were not considered) in the main diagnosis, concomitant diagnoses, diagnostic or therapeutic procedures. The first hospitalisation in this time range was defined as index date. Subjects with a previous ASCVD event or under any LLT drugs, including statins (e.g., ATC code C10AA), ezetimibe (C10AX09), bempedoic acid (C10AX13), lomitapide (C10AX14), mipomersen (C10AX15), inclisiran (C10AX16), and fixed combinations (C10BA, C10BX), in the 2 years prior to the index date were excluded.

Each selected patient was followed for 3 months from the index date, to investigate whether patients received a first LLT prescription (ATC codes shown above); in sensitivity analyses, the follow-up period was extended to 6 and 12 months. Subjects who experienced a second event within the 3 months (6 and 12 months in sensitivity analyses) of follow-up or died during this period were excluded from the selection.

### 2.3 Patient characteristics

The information on several potential confounding factors was collected at the index date or in the 6 months before the index date. In more detail, age and sex were assessed at the index date, as well as the type of event that caused hospitalization (cardiovascular or cerebrovascular event), while the use of antiplatelet agents (e.g., aspirin, clopidogrel; ATC B01), antihypertensives (e.g., diuretics [C03], beta-blockers [C07], calcium channel blockers [C08], ACE inhibitors and ARBs [C09], and centrally acting agents [C02]), antidiabetic drugs (e.g., metformin; ATC A10B), antidepressants (e.g., SSRIs and tricyclics; ATC N06A), and thyroid hormone replacement therapy (e.g., levothyroxine; ATC H03A) (specifically, a subject was considered exposed to one of these drugs only if there were at least two prescriptions dispensed prior to the index date; otherwise, the individual was considered unexposed), and the prevalence of polypharmacy (Masnoon et al., 2017), calculated as the highest number of medications dispensed in any single quarter (i.e., three-month period) during the 6 months prior to the index date. Polypharmacy was defined as the prescription and dispensing of 5 to 9 distinct drug substances within the same quarter, while excessive polypharmacy was defined as 10 or more drug substances within a quarter. Finally, the multisource comorbidity score (MCS) (Corrao et al., 2017) was also calculated including 34 conditions recorded in the year prior to the index date. The MCS index has been divided into the following comorbidity classes: 0–4 diseases, 5–9, ≥10.

### 2.4 Statistical analysis

Categorical variables were compared by chi-square or Fisher’s exact tests. The Kolmogorov-Smirnov test was used to assess the distribution of continuous variables. Variables with normal distribution are shown as mean and SD and were compared using t-test. Variables with skewed distribution are shown as median and interquartile range and were compared using Mann-Whitney.

We assessed the percentage of subjects who initiated an LLT following an ASCVD event within 3 months. In a sensitivity analysis, we then compared rates with those of similar cohorts of subjects enrolled in the same months in the years 2019, 2020, and 2021. The main analysis was stratified by sex, age classes (40–50, 51–60, 61–70, 71–80, >80 years), and type of event to assess if pattern use varied among strata.

A multivariate logistic regression analysis was conducted to assess the impact of several variables (type of event [“cerebrovascular” as reference], sex [“male” as reference], age [“61–70” as reference], antiplatelet, antihypertensive, antidiabetic, antidepressants, and anti-hypothyroidism treatments [“unexposed” as reference], Multisource comorbidity score [“0–4 conditions” as reference], and polytherapy [“total number of different medications by ATC 4th level code <5” as reference]) on the likelihood of initiating treatment. Model estimates are presented as odds ratios (OR) and the corresponding 95% confidence interval (95%CI).

Finally, we assessed the benefit of initiating an LLT within 1 year of the first hospitalization after an ASCVD event, evaluating the all-cause mortality rate.

As an exploratory analysis, we estimated the incremental cost per avoided death, based on average healthcare expenditures during one-year follow-up (including cardiovascular medications and cardiology outpatient visits). Cost calculations were based on Italian NHS reimbursement tariffs (Ministry of Health Decree, 2012) and the ex-factory price of treatments (Gallery - Farmadati, 2024).

Data analysis was performed using SAS (Statistical Analysis System) software version 9.4 (SAS. Institute, Inc. Cary, North Carolina), and two-tailed p-value <0.05 was considered for statistical significance in all analyses.

## 3 Results

The analysed cohort comprised 16,025 individuals with a hospitalization for ASCVD event, between January 1, 2022, and September 30, 2022. Overall, 58.86% of the cohort initiated an LLT after the ASCVD event, with a median time of 9 days post-event (interquartile range: 5–20 days). Temporal trend analysis reveals that the percentage of those treated with an LLT seems to show a growing trend over time (Supplementary Figure S1, p-value for trend <0.0001).

Patients who did not initiate an LLT within 3 months after the ASCVD exhibited a higher percentage of females (44.36% vs. 30.41%, p-value <0.0001) and a higher mean age (mean ± SD 74.10 ± 13.10 vs. 68.52 ± 12.06, p-value <0.0001) (Table 1).

**TABLE 1:** Characteristics of the selected cohort at the enrolment.

Covariates	Total	Lipid-lowering therapy within 3 months after the ASCVD event		p-value
		No	Yes	
N	16,025	6,592	9,433	
Female sex, %	36.15	44.36	30.41	<0.0001
Age, mean (SD)	70.82 (12.80)	74.10 (13.10)	68.52 (12.06)	<0.0001
Age class, %				
40–50 yy	6.61	5.58	7.34	<0.0001
51–60 yy	17.32	11.85	21.14	
61–70 yy	22.99	18.45	26.16	
71–80 yy	26.45	26.67	26.29	
>80 yy	26.03	37.45	19.07	
Cardiovascular event, %	64.46	52.37	72.90	<0.0001
Antidiabetic drugs, %	8.50	7.99	8.85	0.06
Antihypertensive drugs, %	47.58	46.71	48.18	0.07
Antithrombotic drugs, %	20.62	26.96	16.19	<0.0001
Thyroid hormone replacement drugs, %	3.21	3.49	3.02	0.10
Antidepressant drugs, %	7.11	8.68	6.01	<0.0001
Multisource comorbidity score, %				
0–4 conditions	84.51	78.35	88.82	<0.0001
5–9 conditions	11.76	15.94	8.83	
10–14 conditions	2.70	4.19	1.66	
≥15 conditions	1.03	1.52	0.69	
Polytherapy, %				
0–4 drugs	67.69	62.36	71.42	<0.0001
5–9 drugs	24.62	26.61	23.24	
≥10 drugs	7.68	11.03	5.34	

The percentage of subjects starting LLT varied significantly by factors such as sex and age (Table 2). Females had a lower prevalence than males (49.53% vs. 64.15%, p < 0.0001). The highest prevalence was observed in individuals aged 51–60 (71.86%), while it dropped below 50% in those over 80. Interestingly, a statistically significant difference emerges in the percentage of those initiating an LLT depending on whether the event was cardiovascular or cerebrovascular: in the former case, the percentage was 66.58%, compared to 44.87% for cerebrovascular events (p-value <0.0001).

**TABLE 2:** Percentage of the cohort starting a lipid-lowering therapy within 3 months from the index date in the overall cohort and in subgroups by sex, age, and year of enrolment.

	Year of enrolment: 2022; N = 16,025	
	Prevalence, %	p-value
Overall	58.86%	
Stratified by sex		
Male	64.15	<0.0001
Female	49.53	
Stratified by age group		
40–50 yy	65.28	<0.0001
51–60 yy	71.86	
61–70 yy	66.99	
71–80 yy	58.52	
>80 yy	42.15	
Stratified by type of event		
Cardiovascular	66.58	<0.0001
Cerebrovascular	44.87	

In the sensitivity analysis at 6- and 12-month follow-up, the primary result was confirmed, showing only a slight increase in the proportion of patients initiating an LLT after the ASCVD event: 61.91% out of 15,053 hospitalizations at 6 months and 65.01% out of 13,912 hospitalizations at 12 months.

Among those who initiated treatment (Supplementary Table S1), the most prescribed monotherapy was atorvastatin, accounting for 68.75% of the total treatments. Other statins, such as rosuvastatin and simvastatin, were also utilized but at significantly lower frequencies. Combination therapies involving statins and ezetimibe were prescribed in 14.32% of cases. PCSK9 inhibitors (evolocumab and alirocumab) were used less frequently, together representing only 0.27% of prescriptions.

**FIGURE 2:**
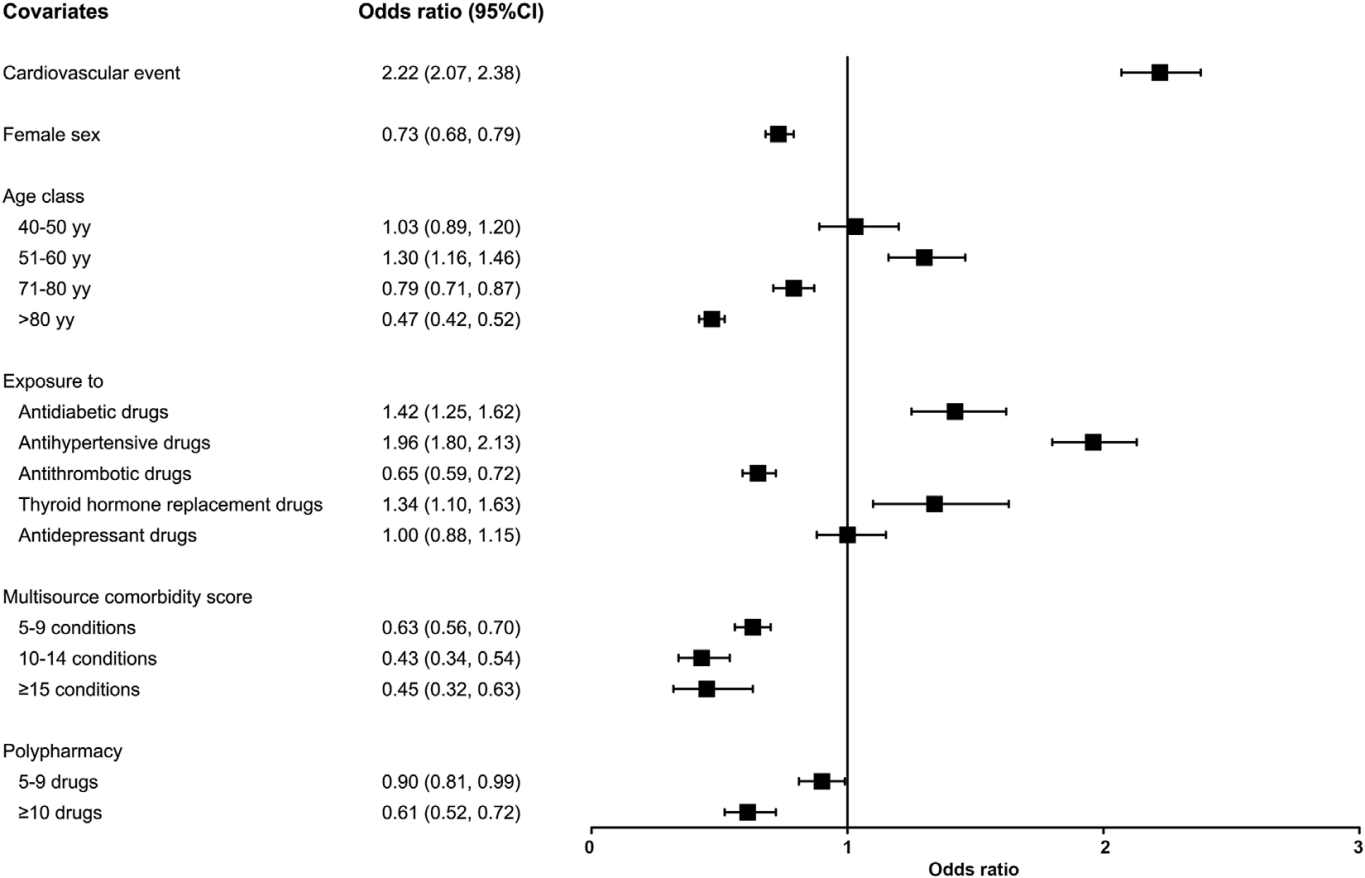
Probability of initiating treatment after an ASCVD event. Association between covariates and the likelihood of initiating treatment (odds ratios [OR] and 95% confidence intervals [95%CI]).

Figure 2 shows the adjusted OR values and 95% CI for the probability of starting the treatment after an ASCVD event. The likelihood of receiving an LLT was higher in subjects who experienced a cardiovascular event (OR 2.22, 95%CI 2.07–2.38) compared to those who experienced a cerebrovascular event, in patients aged 51–60 years (OR 1.30, 95%CI 1.16–1.46), and in patients previously treated with antidiabetic (OR 1.42, 95%CI 1.25–1.62), antihypertensive (OR 1.96, 95%CI 1.80–2.13), and thyroid hormone replacement drugs (OR 1.34, 95%CI 1.10–1.63). Conversely, age 71–80 years (OR 0.79, 95%CI 0.71–0.87), age >80 years (OR 0.47, 95%CI 0.42–0.52), female sex (OR 0.73, 95%CI 0.68–0.79), previous exposure to antithrombotic medications (OR 0.65, 95%CI 0.59–0.72), and polypharmacy (OR 0.90, 95%CI 0.81–0.99 for 5-9 medications; OR 0.61, 95%CI 0.52–0.72 for ≥10 medications) were associated with a lower likelihood of initiating treatment after the event. Also, having a more severe health status, as identified through the MCS score, determines a lower likelihood of initiating treatment, in a dose-dependent manner.

Among those who initiated an LLT after the index event, 316 individuals (3.07%) died within 1 year, compared to 667 individuals (11.66%) among those who did not initiated treatment (p-value <0.0001), with a number needed to treat (NNT, 1/Absolute Risk Reduction) to prevent one death out of 12. Considering an average cost per treated patient of €359.90 compared to €137.64 (Δ = €222.26), we estimated an incremental cost per avoided death of €2,667.10 (Δ*NNT) (Supplementary Table S2).

## 4 Discussion

This study presents updated real-world data regarding the initiation of an LLT among a large Northern Italian cohort of patients discharged from hospital after an ASCVD event. The latest European Society of Cardiology (ESC) and European Atherosclerosis Society (EAS) guidelines (Mach et al., 2020; Byrne et al., 2023) strongly recommend an LLT in ASCVD patients, emphasizing the relevance of early initiation of high-intensity LLT to mitigate cardiovascular risk. However, our analysis confirms that only about 60% of subjects following discharge after an ASCVD event received an LLT. Despite the upward trend observed in the temporal analysis, this percentage remains largely suboptimal.

Evidence collected in our study is consistent with previous real-world data, highlighting unmet treatment needs. In a US analysis of Medicare data in 2014, statin use was 43.0% among those with cerebrovascular disease and 51.7% among those with coronary heart disease (Colantonio et al., 2020), with another analysis showing an increase from 50.3% in 2007 to 59.9% in 2016 (Yao et al., 2020). An observational study by Arca et al. (2018) noted that, in 2015, only 39% of ASCVD patients received any LLT in Italy, of whom only 12% had a high-intensity prescription. In a retrospective cohort study utilizing electronic medical record data from five health systems participating in the CardioHealth Alliance in 2021, out of 75,352 patients with ASCVD not prescribed any LLT at baseline, only 21% were initiated on LLT within 6 months (Shah et al., 2024).

The 2019 ESC/EAS guidelines advocate for a minimum 50% reduction in LDL-C and LDL-C goal of <1.4 mmol/L (<55 mg/dL) for very high-risk individuals. These ambitious goals often require the use of high-intensity monotherapies or combination therapies, which are, however, frequently underutilized. Recent data from the SANTORINI study, a survey conducted in 2020 across 14 European countries, showed that only 2 out of 3 patients with ASCVD were treated with high-intensity LLT or combination therapies, and that 73% failed to meet LDL-C guidelines goals (Ray et al., 2023). Our study presented a more optimistic perspective: among patients who received LLT within 3 months after discharge, nearly more than 90% started with atorvastatin or rosuvastatin monotherapy or with a statin + ezetimibe combination.

As reported in other studies (Colantonio et al., 2020; Yao et al., 2020; Citarella et al., 2022), we confirmed that developing a cardiovascular event was the most influential factor increasing the probability of receiving an LLT prescription following discharge. Conversely, patients discharged after non-hemorrhagic stroke or transient ischemic attack were less likely to receive an LLT. This evidence is in contrast with AHA/ASA guidelines (Kleindorfer et al., 2021), which report that statins are beneficial for preventing recurrent stroke, as well as with a recent meta-analysis (Yin et al., 2022), which showed that statin therapy significantly reduced the risk for ischemic stroke in the ischemic stroke survivors. Moreover, the role of LLT, and statins in particular, in patients with peripheral artery disease (PAD) has been recently highlighted in a Joint Statement by European Atherosclerosis Society/European Society of Vascular Medicine (Belch et al., 2021), reporting that lowering LDL-C not only reduces cardiovascular events but also major adverse limb events, including amputations. In patients with prior ischemic stroke, PCSK9 inhibitors added to statins reduced cardiovascular events by 15%, consistent with benefits seen in patients with MI or PAD (Giugliano et al., 2020).

In accordance with previous studies (Rodriguez et al., 2016; Mazhar et al., 2022), we also confirmed the role of sex and age as influencing factors. We found that women have approximately 27% lower odds than men to be treated with any LLT, consistently with previous observations (Rosenson et al., 2017; Navar et al., 2023). This can be explained both by a tendency among physicians, well-documented in the literature, to prescribe LLT—particularly high-intensity ones—less frequently to female patients (Lopez Ferreruela et al., 2024), as well as by a lower propensity for women to initiate and continue therapy (Olmastroni et al., 2020). In both cases, two factors have been consistently reported in published studies: the belief that women have a lower cardiovascular risk compared to men (Bairey Merz et al., 2017), and the higher incidence of adverse effects of LLT (especially statins) in female patients (Casula et al., 2021). Although the prevalence of ASCVD is higher in men, ASCVD mortality is higher among women, and there is no evidence that an LLT could be less effective for secondary prevention (Baigent et al., 2005).

Compared to patients aged 61–70 years, subjects aged 71–80 and >80 years old were less likely to receive an LLT, conversely to what was observed in younger patients. Similarly, a cohort study (Thalmann et al., 2023) showed that patients aged ≥70 years were consistently less likely to receive statin treatment. Although LLT prescription in the very old patients remains controversial in primary prevention (Cobos-Palacios et al., 2021), the advantages of statin therapy in older adults diagnosed with ASCVD are well recognized. In fact, statin therapy is generally recommended for this age group, except for individuals experiencing severe health impairments, extreme frailty, polypharmacy, or with a limited life expectancy (Cholesterol Treatment Trialists, 2019). Indeed, our observations also suggest that individuals who present concomitant treatments or comorbidities have a lower likelihood of initiating the treatment. Evidence suggests that physicians are reluctant to treat these patients, and that educational activities are needed to promote an appropriate approach to cardiovascular prevention even at older ages.

The relevance of promoting an appropriate approach to prescribing LLT after an ASCVD event is well demonstrated by the evidence of subsequent reductions in morbidity and mortality (Klungel et al., 2002; Han et al., 2022). In our cohort, timely treatment led to a large reduction in all-cause mortality, underscoring the critical role of early intervention in enhancing the survival of high-risk patients. While the implementation of cardiovascular prevention measures inevitably incurs costs (Han et al., 2022), these expenditures should be viewed in the context of their overall health benefits. Our analysis estimated an incremental cost per avoided death of €2,667.10, a figure that reflects both the clinical effectiveness of LLT and its economic sustainability (Michaeli et al., 2023). This relatively modest cost highlights the efficiency of LLT in delivering substantial health gains, particularly in high-risk populations where the potential for preventing adverse cardiovascular outcomes is greatest.

Although our primary focus was on treatment initiation, it is important to acknowledge the crucial role of adherence in ensuring the long-term effectiveness of lipid-lowering therapy (Basios et al., 2025). In the context of secondary prevention, initiating therapy represents only the first step; sustained adherence is essential to achieving meaningful risk reduction and improving clinical outcomes. Poor adherence may undermine the benefits of even the most effective therapies, highlighting the need for strategies that support long-term treatment persistence.

Our study is among the few to highlight the factors influencing the tendency to initiate LLT in patients following hospitalization for ASCVD events who were not previously treated. However, we have to acknowledge several limitations, mainly due to the type of data sources. First, administrative databases do not collect clinical data, which prevented us from evaluating the patient’s cardiovascular risk or to understand any specific reasons for the lack of LLT prescription. Due to the lack of clinical data, we could not fully adjust for important confounding factors, such as lipid levels, which limits our ability to rule out confounding by indication. Second, our data sources do not discriminate whether the absence of treatment was a choice of the clinician or negligence attributable to the patient in the redemption of the prescription. Third, the lack of specific-cause mortality information must be recognised. We used all-cause mortality as a pragmatic and objective outcome, which is particularly suitable in large population-based studies relying on administrative databases, where information on the underlying cause of death may be incomplete or inconsistently recorded. Nevertheless, administrative databases themselves are an element of strength, as they collect all the reimbursed drugs dispensed to all citizens covered by the NHS. Moreover, administrative data collection, managed at a regional level, is nationally standardized, extremely accurate, and routinely used for drug utilization and pharmacoepidemiologic research (Trifiro et al., 2019). Finally, regarding the economic evaluation, we had only partially considered direct costs, this might lead to an underestimation. However, our objective was to demonstrate that, regardless of the cost required to prevent a death, there is a clear benefit in treating patients after an ASCVD event, as their mortality rate is significantly lower compared to untreated individuals.

In conclusion, our study reported that LLT prescription, especially regarding high-intensity treatments, remains suboptimal in patients discharged after an ASCVD event, suggesting unmet needs among these patients in the contemporary real-world setting. A more comprehensive understanding of the reasons beyond failure to start treatment or therapeutic discontinuation is crucial to identify actual barriers that should be addressed, ultimately leading to changes in practice that could translate to improved adherence to guidelines. Further educational interventions, as well as monitoring strategies at patient and hospital levels, could be implemented to enhance LLT rates following discharge for an ASCVD event.

## Data Availability

The data used in this manuscript are property of the Lombardy Region and cannot be shared directly by the authors. Access to these data can only be obtained through formal request to the data owner, in compliance with regional regulations.
