# Supplementary material for: Predictors and benefits of lipid-lowering therapy initiation after an atherosclerotic cardiovascular event: a retrospective cohort study
Source: Front Pharmacol. 2025 Sep 25;16:1588376. doi: 10.3389/fphar.2025.1588376 (PMC12507633; doi:10.3389/fphar.2025.1588376)
Supplement: Supplementary file 1 [file Supplementaryfile1.docx]

Supplementary Material

**Supplementary Figure 1.** Temporal trend analysis for the percentage of patients starting a lipid-lowering therapy after an ASCVD event.


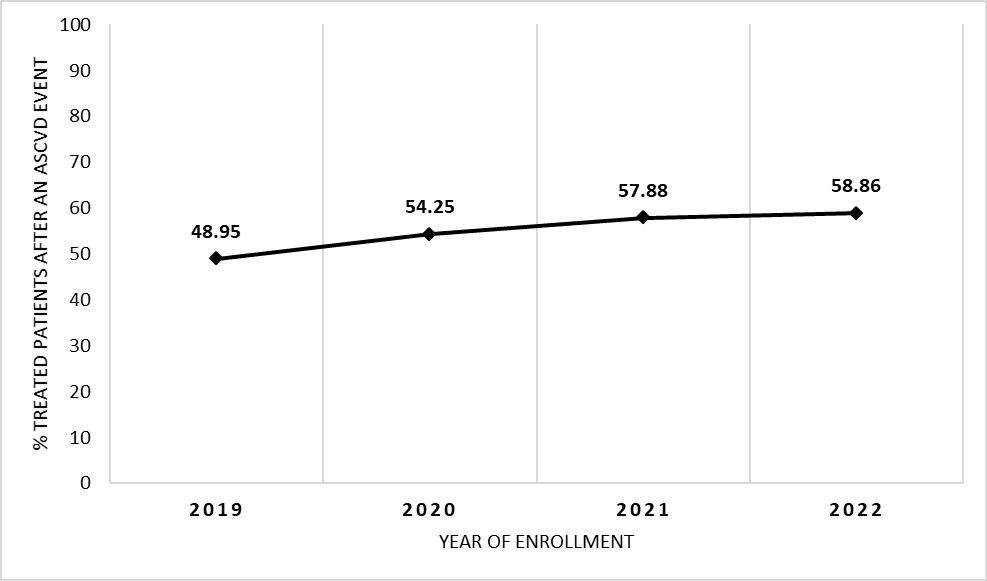


**Supplementary Table 1.** Description of the first lipid-lowering drug prescribed after an ASCVD event among those who initiated treatment within 3 months from the index date.

|  |  | **Year of enrolment: 2022** | |
| --- | --- | --- | --- |
| **ATC code** | **Generic drug name** | **N** | **%** |
| C10AA01 | Simvastatin | 265 | 2.81 |
| C10AA02 | Lovastatin | 5 | 0.05 |
| C10AA03 | Pravastatin | 6 | 0.06 |
| C10AA04 | Fluvastatin | 1 | 0.01 |
| C10AA05 | Atorvastatin | 6485 | 68.75 |
| C10AA07 | Rosuvastatin | 1009 | 10.70 |
| C10AX09 | Ezetimibe | 286 | 3.03 |
| C10AX13 | Evolocumab | 16 | 0.17 |
| C10AX14 | Alirocumab | 9 | 0.10 |
| C10BA02 | Simvastatin and ezetimibe | 39 | 0.41 |
| C10BA05 | Atorvastatin and ezetimibe | 148 | 1.57 |
| C10BA06 | Rosuvastatin and ezetimibe | 1164 | 12.34 |

**Supplementary Table 2.** Costs associated with cardiovascular prescriptions and cardiology outpatient visits during the one-year follow-up after the index event among treated and untreated patients.

|  | **Total costs** | |
| --- | --- | --- |
|  |  |  |
|  | LLT initiated before  the fatal event | LLT never initiated |
| N | 10303 | 5722 |
| Pharmacological treatments | 3,573,423.98 € | 762,778.17 € |
| Cardiology outpatient visits | 134,599.90 € | 24,792.00 € |
| **Total** | 3,708,023.88 € | 787,570.17 € |
| **Mean cost per patient** | **359.90 €** | **137.64 €** |

**Pharmacological treatments: lipid-lowering treatment (C10AA, C10AX13, C10AX14, C10AX17, C10BA01, C10BA02, C10BA03, C10BA04, C10BA05, C10BA06, C10BA07, C10BA08, C10BA09, C10BA11, C10BA12, C10BX, C10AX09); antiplatelet treatment (B01); antihypertensive treatment (C02, C03, C07, C08, C09); antidiabetic treatment(A10B).*
